# Aortic Disease: Barbiturate Poisoning

**Published:** 1965-04

**Authors:** T. F. Hewer


### AORTIC DISEASE: BARBITURATE POISONING

*A Clinico-Pathological Conference held in the University of Bristol on 3rd November 1964*

CHAIRMAN: PROFESSOR T. F. HEWER

*Dr. Barritt:* I first saw this lady in 1962 in Ham Green hospital where she had been admitted with heart failure. It was clear that her heart failure was due to aortic valvular stenosis. She gave a history of having a heart murmur nearly all her life. The physical signs were that the left ventricle was hypertrophied and forceful and there was a very harsh systolic murmur, with a palpable thrill over the aortic area and there was an immediate diastolic murmur to be heard at the left sternal edge. I do not think the pulse was particularly abnormal and I suppose this was because she had a combination of aortic stenosis and incompetence. The electrocardiogram showed gross left ventricular hypertrophy. Later we had radiological evidence of calcification of the aortic valve. So there was never any doubt that she had aortic valvular disease. She did not give a history of having had rheumatic fever. I regarded it as likely that she had congenital anomaly of the aortic valve which had become calcified.

She was next admitted to the B.R.I. in June 1962 when her principal complaint was that she had been made very uncomfortable by palpitations. The symptoms of her aortic stenosis had previously been heart failure with breathlessness and some swelling of the ankles and sometimes central chest pain on exertion, in other words angina. On this occasion she was miserable because she had had very frequent attacks of uncomfortably rapid heart action due to paroxysmal tachycardia. When she first came in the electrocardiogram showed very frequent ventricular extra systoles and then there would be times when, for several minutes, she had very rapid regular heart rate, about 160/minute, and the electrocardiogram showed ventricular tachycardia lasting for a minute or two. She felt faint and very unhappy when this was happening and she was having continual attacks.

There was not much that we could do about it. When I saw her on the first occasion I thought that probably she was overdigitalized and the ventricle was being stimulated to give off frequent ectopic beats and so we took her off digitalis for a couple of days, then started her again on half the dose. This did not really help her. We also gave her procaine-amide, which is a fairly powerful myocardial depressant and is sometimes quite successful in diminishing the frequency of ventricular ectopic beats. While she was in the ward on procaine-amide she was reasonably comfortable and we sent her home on this drug and I did not see her again. Her doctor talked to me over the telephone about her several times. She had a period when she was relatively free of paroxysmal tachycardia and then developed it again. About six months after she had left the ward he telephoned me to say that she had had hemiplegia, could this be due to cerebral embolism? I did not think that there was any good reason why she should get cerebral embolism because she did not have auricular fibrillation and therefore there would be no strong tendency for her to form thrombi in the atrium, and nothing suggested that she, at any rate when we had seen her, had bacterial endocarditis. She was of course 64 years old by this time and clearly the lesion of the brain could simply have been cerebral thrombosis.

This was the last I heard of her. She in fact came as an emergency in May 1964, under Professor Perry. She was deeply unconscious on admission and there was a story that she had been found unconscious at home by her daughter-in-law, and by her was a note saying that she had taken 50 tablets. I ascertained from her doctor that the tablets she had taken were pentobarbitone and heptobarbitone. The story was that she had been depressed by the disability which she suffered from her stroke. She had obviously swallowed a very large number of tablets. She had rather a low blood pressure and in a very few hours she was dead by barbiturate poisoning.

Before she died a sample of blood was taken so that the barbiturate level could be estimated and this gave a very high level of over 4 mg per cent.

So from the cardiological point of view, here is a patient who for a long time had a murmur and all the physical signs of severe aortic valve disease. In spite of her attack of congestive heart failure and many attacks of paroxysmal tachycardia she survived three to four years and finally died of barbiturate poisoning.

*Professor Neale:* Was there any hint in the history of the aetiology of the aortic valve disease?

*Dr. Barritt:* No, there was just nothing to tell us why she had it.

*Professor Hewer:* Did you say the aortic valve was calcified?

*Dr. Barritt:* There were X-rays taken which showed calcification in the region of the aortic valve and I think it would be extremely surprising to find aortic valve disease at that age without calcification.

*Professor Neale:* She did not give a past history of episodes of syncope?

*Dr. Barritt:* No, she just went rather pale and was terribly uncomfortable, but she did not faint.

*Professor Hewer:* You say the digitalis was given rather intermittently?

*Dr. Barritt:* No, she was having a fairly large maintenance dose of digitalis and I thought when I first saw her that perhaps she was overdigitalized and this was contributing to her arrhythmia so we reduced her dose, but I think this was incorrect.

*Professor Hewer:* So that's why you gave the procaine-amide?

*Dr. Barritt:* Yes. There is no doubt that this did considerably diminish the frequency of her attacks.

*Professor Neale:* Would you like to say a word, before we hear the post mortem findings, about the pharmacology of procaine-amide in this sort of case.

*Dr. Barritt:* I am afraid I know very little about pharmacology. It is a fairly powerful depressant of the myocardium; it is sometimes used intravenously to get a good concentration to try and stop ventricular tachycardia. It used to be used, of course, for the treatment or prevention of tachycardia. If you have a patient, say, who has had a myocardial infarction complicated by rapid ventricular tachycardia, obviously the patient is in danger of dying in the attack and one badly wants to stop it, and before the days of electrical counter-shock this was a standard treatment, to give procaine-amide. If you give it intravenously it often causes quite a considerable fall in blood pressure and is quite capable of causing profound depression of the myocardium, so you can get in trouble with it, but it usually stops the attack.

*Professor Hewer:* You give it just once?

*Dr. Barritt:* Yes. Well, you might then go on to give a maintenance dose.

*Dr. Cornes:* The cause of death in this patient was congestive heart failure. She and marked oedema of the lungs. She had congestion of the liver with prominent vascular markings, congestion of the kidneys, and pitting oedema of the ankles.

The cause of the congestive heart failure was calcific aortic stenosis. When I ran some saline above the aortic valve it all went through easily into the left ventricle.

Plate X shows that the cusp margins of the aortic valve are thickened, the commissures are fused, and in the middle of the valve cusps there are thick calcified masses. The right cusp is smaller and thinner than the anterior cusp or the left cusp, and this suggests that there was some congenital deficiency of this particular cusp.

Plate XI is a mounted section of the aortic cusp and shows calcified masses in the cusp, together with a considerable amount of fibrous tissue. I took sections of different parts of the heart to see if I could find any evidence of rheumatic heart disease, but

I was unable to find any. The left ventricle was very greatly hypertrophied, 2·7 cm in thickness at the base compared with a normal upper limit of about 1·5 cm, and this was reflected in the heart weight, which was 540 g, compared with an expected weight of about 320 g.

In addition to these lesions of the heart there was an old cerebral infarct in the left cerebral hemisphere close to the internal capsule. I could not find the cause for this infarct. There was no mural thrombus anywhere in the heart, in the chambers or on the heart valves, and this patient was remarkably free of atheroma.

I came to the conclusion, since the blood barbiturate in this case was only 4·6 mg per cent, that if this woman had not had severe heart disease she would probably have recovered from the barbiturate poisoning. In the Coroner's Court I gave the cause of death as "Heart failure due to calcific aortic stenosis with incompetence in a person who had taken an overdose of barbiturates".

*Professor Neale:* What did you decide was the cause of the aortic calcification?

*Dr. Cornes:* I am very anxious to hear what Dr. Barritt has to say about this. This is a calcific aortic stenosis and the common causes of this are old rheumatic heart disease, or some congenital defect. She had a congenital defect of the right cusp of the aortic valve which was smaller than the other two, seemed to be poorly formed, and I just wonder whether the basis of this calcific valve disease is in fact this congenital defect or whether Dr. Barritt would think that this was possibly a case of rheumatic heart disease.

*Professor Hewer:* Did you say the mitral valve was normal?

*Dr. Cornes:* Yes, sir. All the other valves were normal.

*Dr. Barritt:* I do not know what is the matter with the valve, but my guess would be that the top commissure, which has fused in nearly the centre of the valve orifice, has been like that since the patient was born.

If you look at Leonardo da Vinci's drawings of possible valves, he worked it out very convincingly. He showed that if you have four cusps the thing is unsound from an engineering point of view and is likely to regurgitate, but if you have three cusps they are so formed that they will open so that there is practically no obstruction to forward flow and their ability to withstand strain when they close is maximal. That is what he said and he made a very nice story of it.

Now if in this case it is true that this commissure has been fused from birth then during ejection there has always been some obstruction to ventricular emptying. My guess is that during rapid ejection the cusps would probably vibrate and I think the whole business of turbulence there probably leads to the accretion of platelets, fibrin and, over 60 years, a mass of calcium. That, I would guess, is the most likely explanation.

*Professor Neale:* May I support all that. I think this is the best explanation of this that we have had in this room for several years, and gets us out of the cyclical argument as to rheumatism or not.

I think we must now agree with Dr. Barritt that this is a basic condition of congenital malformation of the aortic valve and of course it could have been recognized earlier in her life. This might have been an ideal case for surgical replacement of the aortic valve. However, like some other cases of congenital heart failure, she lived for 60 years without any serious breakdown.

*Dr. Barritt:* Obviously the only thing you can do with that valve is to cut it out and try to put a better one in its place. But if you think about this woman's life history it makes you a little hesitant about whether we ought to recommend this. After all this patient was reasonably well until the age of 60 at any rate, and thereafter lived quite a time. I think it is true to say that no one putting in a prosthesis in the year 1964 would have any great confidence that what he puts in would do any better than, probably not as well as the original valve.

*Professor Neale:* Did you say the coronary vessels were affected in any way?

*Dr. Cornes:* No, they were quite normal.

*Professor Hewer:* Were the origins of the coronary arteries blocked in any way?

*Dr. Cornes:* The coronary arteries were in fact hypertrophied and no obstruction could be found.

*Dr. Barritt:* That is interesting to know. She did in fact have angina pectoris. Again, another of one's worries when advising operation on patients with this trouble is if they have angina you are always worried that they may have coronary disease. Many of them do. Some of them don't. It is nice to know that you can get anginal pain with perfectly normal coronary arteries.

*Professor Neale:* As a matter of clinical interest, Dr. Barritt, would you like to tell us where the apex beat was?

*Dr. Barritt:* I cannot tell you. I would not have written it down. This is a thing we discuss very frequently in the ward. I don't think you ever see cardiologists counting rib spaces or measuring from the midline. What everybody does, I think, is to feel the apex beat, looking at the chest, and decide whether it is in fact displaced or not. There is not really an adequate measurement. You see students are still told to say that the apex beat is on one or other side of the mid-clavicular line. This is perhaps not the right place to talk about it, but there isn't any such line. Nobody has ever measured the middle of the clavicle and drawn a line down the middle of the patient and drawn a parallel line.

*Professor Neale:* We've got another Leonardo amongst us! Looking at this woman's chest when she perhaps was slightly uncomfortable, would you have noticed an abnormal cardiac impulse?

*Dr. Barritt:* I think almost certainly, yes.

*Student:* What treatment was given for the poisoning?

*Dr. Barritt:* I don't think I can answer the question very well, there isn't very much written in the notes about it. I am sure that the principal treatment would have been directed towards making sure that she was well ventilated and that she did not have any sort of obstruction to her airway. I think really that is the only treatment of barbiturate poisoning that there is, apart from taking deliberate steps to wash the barbiturate out of the bloodstream. I have no doubt that great care was taken to see that her airway was adequate and that she was ventilating well. I do not suppose there was anything else that was or could have been done.

*Dr. Cornes:* I would just like to know at what level of blood barbiturate does one get worried? At what level does one say "this person is not likely to recover"? I don't know the answer.

*Dr. Barritt:* I don't think anyone else does either. I think the usual clinical guide is that if you have a high level, and this level of 4·6 mg per cent is well above the level that might make a patient unconscious, the guide is really how fast it falls. If you found in a few hours that the level was the same, or higher, or not very much lower, then you would probably feel you should take steps to wash it out. I am not an expert on this. I do not think I can answer the question very well, but what you want is evidence that the level is going down.

*Student:* Have analeptics gone out in the treatment of barbiturate poisoning?

*Dr. Barritt:* I think I can speak for fashion all right. Yes, they have gone out nearly completely. In the old days we used to be giving picro-toxin every few minutes to make the patient convulse. This has gone out of fashion completely. You will remember that another drug, Megimide, was brought in, and this was supposed to be superior, but this also has completely gone out of fashion and I think has rightly been superceded by artificial removal of barbiturates in the blood in a patient who is obviously not recovering of his own accord.

*Dr. Hunt:* Is not the answer to Dr. Cornes' question: if they are alive they still might recover and there is no such thing as a level at which they are never going to recover?

*Dr. Barritt:* I don't suppose there is. Some patients die of barbiturate poisoning, but I do not suppose anyone yet knows what the damage to the brain is due to. It may simply be due to a period of very poor ventilation and very severe hypoxia. Whether the drug itself is a cerebral metabolic poison and ruins the cell, I don't think anybody knows.

*Dr. Norman:* I only know the commonest cause of holes in the globus pallidus is either carbon monoxide or barbiturate poisoning.

*Dr. Barritt:* Is it suggested that it is really hypoxic?

*Dr. Norman:* Many people think it may be due to pressure on the vessels from cerebral oedema. I think there must be some direct histotoxic action.

*Dr. Barritt:* Of course you get patients with barbiturate poisoning who don't die until a day or two after they have taken the tablets, and even though the brain is destroyed more or less, they are kept alive for a number of days, and this makes it more difficult to tell what the initial damage to the brain was.

*Dr. Norman:* I wonder if you get delayed effects in patients who survive barbiturate poisoning? It is well known that after carbon monoxide poisoning signs of brain damage may appear several weeks after the patient has made an apparently complete recovery.

*Dr. Enoch:* Could Dr. Barritt tell us about the place of pressor drugs in cases of barbiturate poisoning where there is hypotension of a degree which might jeopardize the renal circulation?

*Dr. Barritt:* No. I talked about the artificial means of removing poison from the blood; I should have said, of course, that one should also make every effort to get the kidneys to do it and that is really what you are asking, isn't it?

I think usually one gives fairly large volumes of fluid intravenously and perhaps a diuretic to get a good flow of urine and hopes the barbiturate will be carried out in that way. I would think that it was really physiologically unsound to give pressor amines because these are likely to reduce renal blood flow even further.

*Dr. Enoch:* Do you not worry about the blood pressure at all?

*Dr. Barritt:* Under these circumstances I would have thought renal blood flow would be more important. If you are dealing with something like cardiogenic shock and myocardial infarction you have a better argument for keeping up central aortic pressure, but I would have thought that under this sort of circumstance you would be likely to lose more than you would gain by constricting the vessels.

*Dr. Cornes:* If a person who has taken barbiturates is unconscious two days later, would you as a clinician expect this person would therefore die?

*Dr. Barritt:* If there is some evidence that they are improving you expect them to recover completely; I mean if the barbiturate level is coming down and they are breathing well I think you would have every expectation that they would recover completely.

